# Purification, concentration and recovery of small fragments of DNA from Giardia lamblia and their use for other molecular techniques

**DOI:** 10.1016/j.mex.2017.08.005

**Published:** 2017-09-08

**Authors:** Fabiola Santos, Saúl Gómez-Manzo, Edgar Sierra-Palacios, Abigail González-Valdez, Adriana Castillo-Villanueva, Horacio Reyes-Vivas, Jaime Marcial-Quino

**Affiliations:** a Escuela Nacional de Ciencias Biológicas, Instituto Politécnico Nacional, 11340, Mexico; b Departamento de Medicina Experimental, Facultad de Medicina, Universidad Nacional Autónoma de México, 04510, Mexico; c Laboratorio Bioquímica Genética, Instituto Nacional de Pediatría, Secretaría de Salud, 04530, Mexico; d Colegio de Ciencias y Humanidades, Plantel Casa Libertad, Universidad Autónoma de la Ciudad de México, 09620, Mexico; e Departamento de Biología Molecular y Biotecnología, Instituto de Investigaciones Biomédicas, Universidad Nacional Autónoma de México, 04530, Mexico; f CONACyT-Instituto Nacional de Pediatría, Secretaría de Salud, 04530, Mexico

**Keywords:** *purification*, *agarose gel*, *small DNA*, *PCR*, *Giardia lamblia*

## Abstract

Purification of nucleic acids is an essential procedure for most experiments in molecular biology. In this paper, the freeze-squeeze method with some modifications is proposed as an alternative methodology for the purification, concentration and recovery of small DNA fragments from agarose gels. The advantage of this alternative methodology is that it enables the recovery of fragments that are less than 100 bp in length and enables suspension of products in smaller volumes compared to several commercially available kits. In addition, the purified fragments were re-amplified by PCR and used for cloning and sequencing. Moreover, this protocol was used to perform the isolation and identification of microRNAs from Giardia lamblia, as previously reported. This protocol has the advantage of being inexpensive and easy and can be employed for various molecular applications. The advantages of this protocol include

• A modified classical method was used for purification of small DNA fragments from G. lamblia.
• The modified freeze-squeeze method was more efficient in cleaning up small DNA fragments from agarose gels compared to commercial kits.
• The modified method allows concentration and recovery of fragments up to 60 bp in length.
• The modified freeze-squeeze method allows re-suspension of the products in volumes of up to 2.5 μL.

## Graphical abstract

**Figure:**
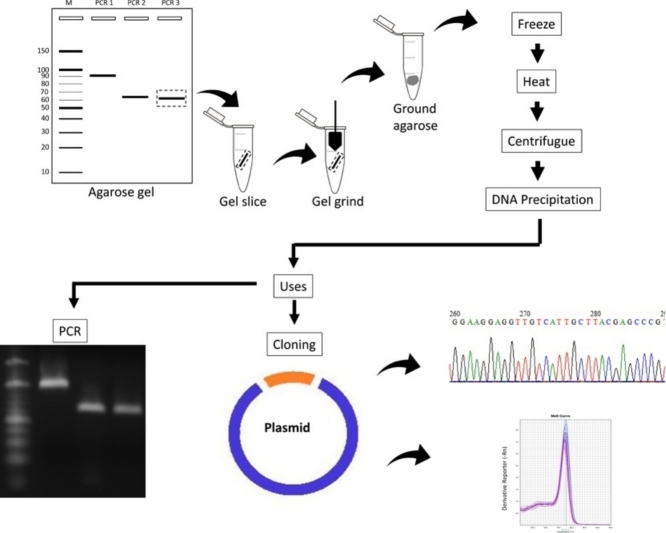

## Method Details

### Overview

Purification of nucleic acids is an essential procedure for most experiments in molecular biology because the presence of impurities can interfere with such molecular applications as restriction digestion, ligation, cloning, labeling sequencing, PCR and RT-qPCR. In order to solve this problem, several methods for purification of DNA fragments have been developed, such as those utilizing phenol-chloroform, paper strip and magnetic particles (for aqueous solutions); other methods include purifying DNA from fragments of agarose gel, known as the freeze-squeeze method which was one of the first methods to be reported. This method has been widely used for DNA purification but has the disadvantage of using low-melting point agarose and/or requiring high concentrations of DNA samples. Moreover, it has been observed that this method gives a better yield for large DNA fragments [1], [2], [3], [4]. Finally, spin columns and dialysis tubing are also used to purify DNA, but these are mainly employed in commercial kits [5], [6]. Because the columns of the kits are made from resins, silica-based membrane or magnetic particles, the cost of products may be high [7]. Furthermore, the kits may have disadvantages. For example, the quality and concentration of DNA recovered depends on the procedure used, and recovery of large and small fragments of DNA may be inefficient [8], [9]. Based on the abovementioned considerations and our needs for purifying and analyzing small fragments of DNA or molecules, such as microRNAs, involved in the genetic regulation in *G*. *lamblia*, we modified a technique previously reported as the freeze-squeeze method to obtain these small fragments and determined the functionality of this modified methodology. Although this technique has been used for many years, there are no reports describing the purification of small fragments of DNA (<100 bp). It is important to emphasize that this proposal only provides an alternative for purification and recovery of small DNA fragments without indicating that it is a better procedure compared to all the existing commercial kits.

### Method

In this work, a methodological strategy is described, which enabled the purification, recovery and concentration of DNA fragments with length <100 bp using gene fragments from *G. lamblia*. To achieve our goal, we used a classic technique of purification, the freeze-squeeze method, with some modifications [1], [2], [3], [8]. Some of the major modifications were that we did not use the traditional glass wool, the tubes were not subject to any type of adaptation and low-melting agarose was not utilized. All procedures used are described below.

*Reagents*

Oligo *TPI* Fw: AGGAGCTCGGAGAGTCCAA

Oligo *TPI* Rv: ACACGGGCTCGTAAGCAAT

Oligo *NADHox* Fw: GCACCATATGGCTTCAACGG

Oligo NADHox Rv: CAGGCCTGTCCGTGTCATTA)

Oligo GlsRNA17 Fw: TGCAGCCTAATCACCGC

Oligo GlsRNA 17 Rv: GTGCAGGGTCCGAGGT

Phusion HighFidelity DNA polymerase (Thermo scientific)

*Xho*I (Thermo Scientific)

*Nco*I (Thermo Scientific)

GelRed (Nucleic Acid Gel, Biotium)

1X TBE buffer (Tris-HCL/Boric Acid/EDTA)

TE buffer [10 mM Tris-HCl, pH 8.0 and 1 mM (ethylenedinitrilo)tetraacetic acid (EDTA)]

Sodium acetate (3 M, pH 5.2)

Ethanol for molecular biology (Sigma-Aldrich)

SyberGreen Master Mix kit (Applied Biosystems, CA, USA)

GeneJET Plasmid Miniprep Kit (Thermo Scientific)

T4 ligase (Thermo Scientific)

Agarose (Invitrogen)

GeneJET Plasmid Miniprep Kit (Thermo Scientific)

*Equipment*

Thermocycler MaxyGene Gradient (Axygen, USA)

Thermomixer compact, Eppendorf

Centrifuge (minispin Eppendorf centrifuge for 1.5 mL microcentrifuge tubes)

Agarose Electrophoresis System (Thermo Scientific)

Analyzer ImageJ1.50i, Wayne Rasband National Institutes of Health (USA)

StepOne™ Real-Time PCR System and Fast SYBR^®^

MultiDoc-It Digital Imaging System UVP

## Purification of small DNA fragments from agarose gels

1. Small DNA fragments obtained by PCR were run on agarose gels and 1X TBE buffer (Tris-HCL/Boric Acid/EDTA). The gels were stained with GelRed (Nucleic Acid Gel, Biotium) and visualized on a MultiDoc-It (UVP). Following electrophoresis, the DNA fragments were extracted from the agarose gel using a clear scalpel or razor blade and the gel slice was placed in a sterile 1.5 mL microcentrifuge tube.
2. The gel slice placed in the tube was ground with the help of a metal rod and subsequently 150 μL of TE buffer [10 mM Tris-HCl, pH 8.0 and 1 mM (ethylenedinitrilo) tetraacetic acid (EDTA)] was added.
3. The gel mixture was quickly frozen using dry ice (liquid nitrogen may also be employed)
4. Next, the tubes were incubated at 72 °C for 3 min in a Thermo block (Thermomixer compact, Eppendorf).
5. Immediately following incubation, the tubes were centrifuged at 10,000 x *g* for 30 s at room temperature (RT).
6. The solubilized gel solution was transferred to a new sterile tube (1.5 mL)
7. The DNA contained in the solution was precipitated by addition of 1/10 vol of sodium acetate (3 M, pH 5.2) and 2.5 vol of cold ethanol (or isopropyl alcohol).
8. The tubes were incubated overnight at −20 °C. Note: The incubation time for the precipitation depends of the initial concentration of DNA in the sample.
9. Thereafter, the tubes were centrifuged at 16,000 x *g* for 20 min at RT
10. Finally, the pellets were washed with 600 μL of 70% ethanol (v/v), dried and re-suspended by adding TE buffer [10 mM Tris-HCl, pH 8.5 and 1 mM EDTA] or water to the desired volume.
11. The DNA obtained by this procedure can be immediately used or stored at −20 °C until further use.

## Method validation

**Fig. 1:**
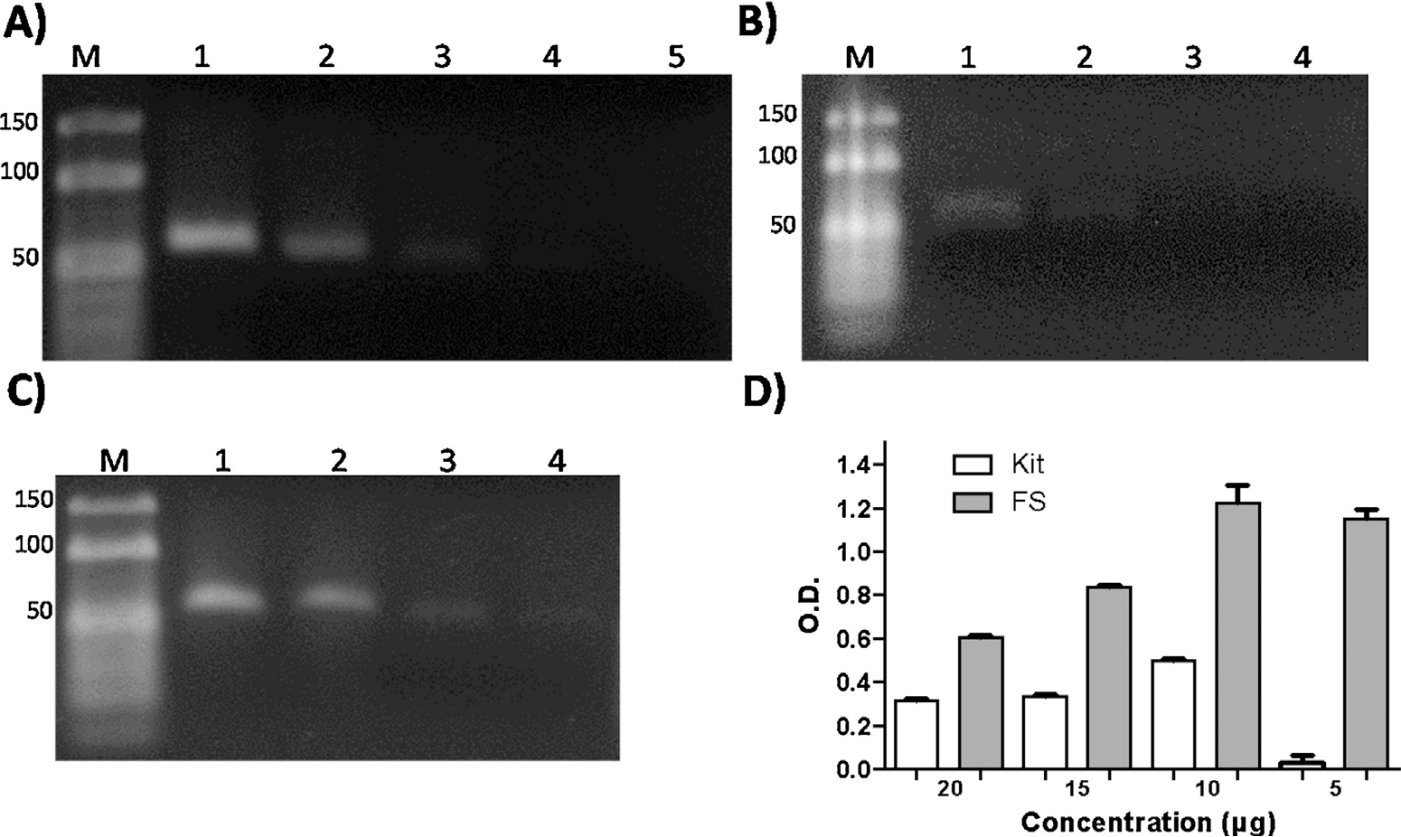
Effectiveness of the purification of small DNA fragments from gel using agarose standard. A) Different concentrations of the PCR products of the *TPI* gene were separated on agarose gel (2.5%). Line 1: 20 μg; 2: 15 μg; 3: 10 μg; 4: 5 μg; 5: 2.5 μg. B) *TPI* gene products purified from agarose gel using the commercial kit. The recovery of the amplicons was only obtained for concentrations of 10 and 20 μg (Line 1 and 2). C) *TPI* gene products purified using the modified freeze-squeeze method. The PCR products were recovered at concentrations from 20 μg to 5 μg (Line 1–4). D) Densitometric analysis of the bands recovered from the gel purified using the commercial kit and the modified freeze-squeeze method. Densitometric analysis of the agarose gels was performed using ImageJ1.50i, Wayne Rasband National Institutes of Health (USA) and the values obtained for each band recovered with both protocols were normalized with their respective concentrations.

We performed several adaptations to the traditional freeze-squeeze method, which allowed the purification, recovery and concentration of DNA fragments from *G. lamblia* with length <100 bp. The need for modification arises from the major obstacles encountered in the purification and recovery of DNA fragments such as their small length, low concentration and diffusion of the bands during the run of electrophoresis. Furthermore, we compared the efficiency of our modified procedure to that of a commercial kit, using different concentrations of the PCR product with an approximate size of 60 bp (Fig. 1A).

Results of the purification of the DNA band extracted from the agarose gel with the commercial kit demonstrated that fragment recovery was possible only at concentrations of 20 μg and 15 μg, respectively (Fig. 1B). In contrast, using our modified freeze-squeeze method, it was possible to purify and recover the desired fragment with at least four different dilutions of the analyzed bands, ∼5 μg being the minimum concentration observed (Fig. 1C). The products purified by both methods (kit and modified freeze-squeeze) were diluted to a final volume of 20 μL of water. Densitometric analysis of the agarose gels demonstrated that the modified method enabled better purification and recovery of the PCR fragments compared to the commercial kit. Therefore, approximately 50% more product was recovered using the modified method, as compared to the commercial kit, at least for concentrations of 20 μg and 15 μg, respectively (Fig. 1D). Our results demonstrated that the limitations of commercial kits used in the purification of small DNA fragments are that their efficiency depends on the concentration of the product, the final elution volume for purification, and the concentration of agarose used for the gel, and many kits specify these requirements in their purification protocols.

**Fig. 2:**
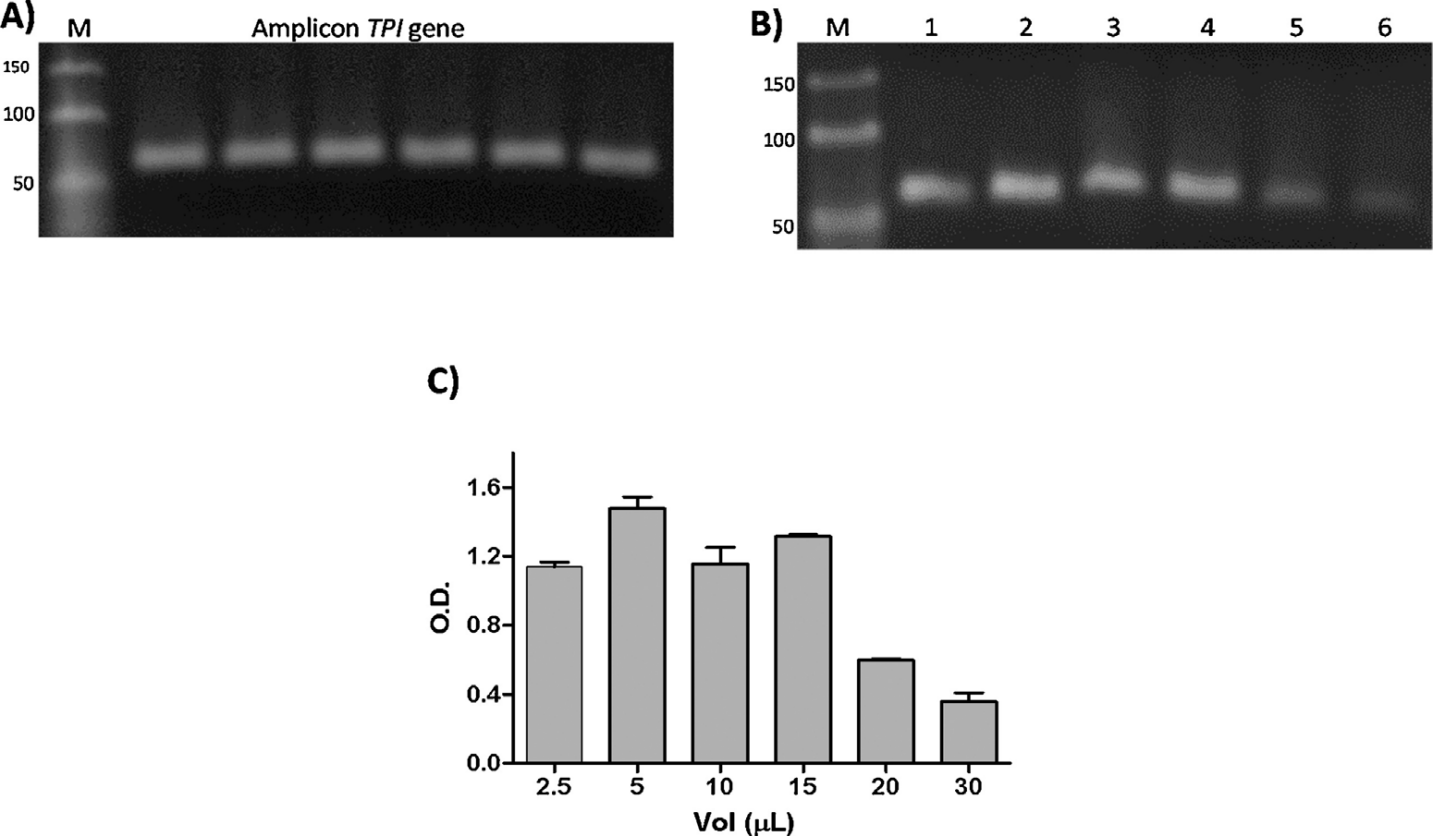
Elution volumes used in the purification of small DNA fragments from agarose gels. A) Amplified fragments of *TPI* gene obtained by conventional PCR and diluted to a concentration of 15 μg. B) Fragments purified from the agarose gel by the modified freeze-squeeze method and re-suspended in different volumes of MilliQ water. C) Densitometric analysis performed for gel containing purified fragments and re-suspended in different volumes of water (2.5 μL, 5 μL, 10 μL, 15 μL, 20 μL and 30 μL).

Per the abovementioned procedure, the PCR product of the *TPI* gene was loaded into an agarose gel using a single concentration (Fig. 2A); following electrophoresis, the bands were extracted from the agarose gel and processed, but now we modified the re-suspension volume of the purified products. The results demonstrated that the analyzed fragment could be recovered and concentrated in a re-suspension volume ranging of 2.5 μL to 30 μL (Fig. 2B). It is interesting to note that we were able to recover the PCR products in a minimum volume of 2.5 μL, which is important for our purposes, whereas with commercial kits it was not possible to re-suspend in a small volume. The densitometry analysis results obtained for the bands in the agarose gel indicate that with the modified method the elution volumes of the samples can be from 2.5 to 15 μL because the DNA concentration is high for these volumes and does not show differences according to the standard deviations (SD) obtained (Fig. 2C). The SD obtained was the result of three experimental replicates. It is noteworthy that in most commercial kits, the minimum elution volume for fragments is 20 μL and depends of the concentration of the fragment, which is a notable limitation. However, in our modified method, the elution volume was not a limiting factor.

**Fig. 3:**
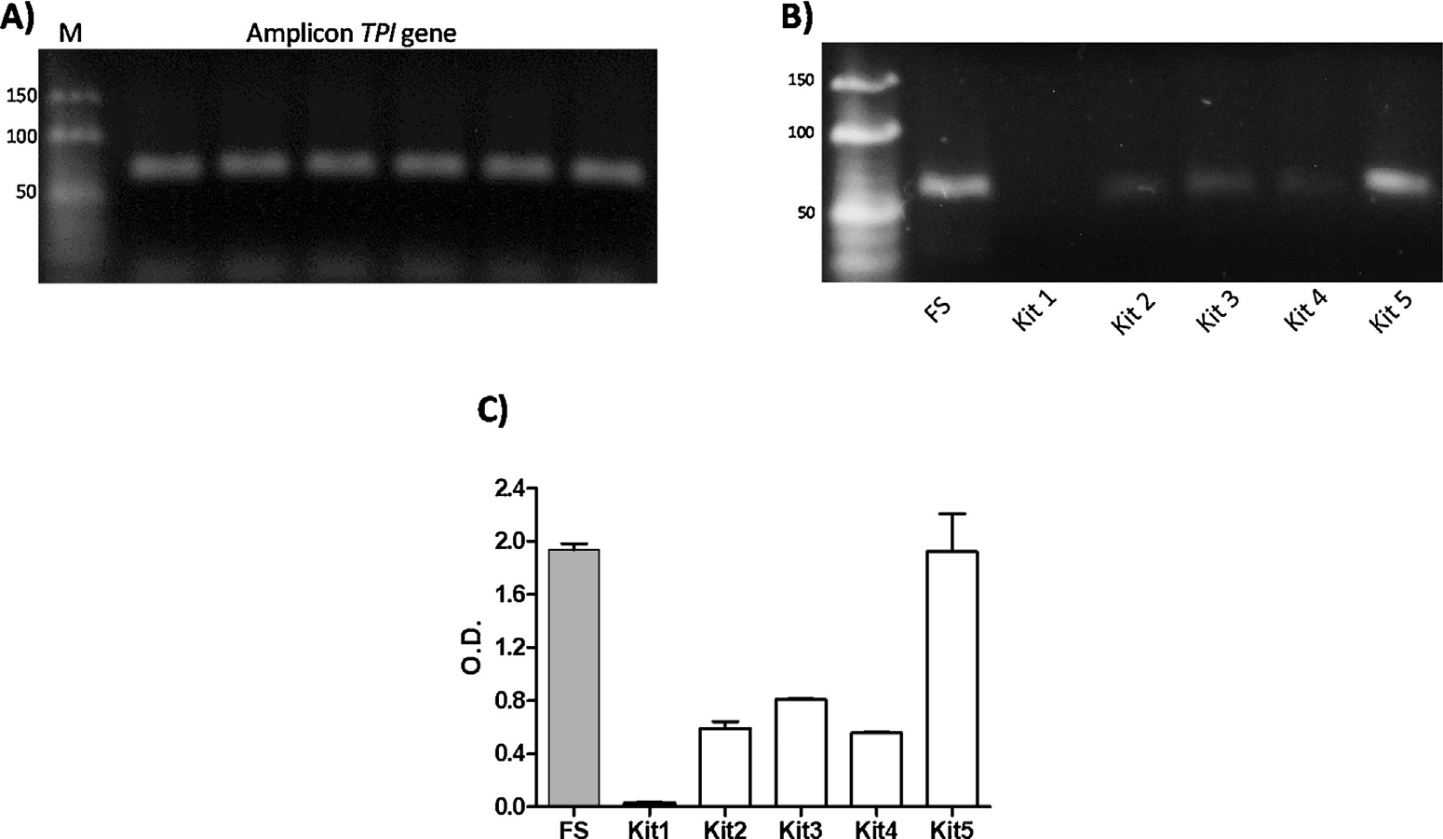
Purification and recovery of small DNA fragments using different gel extraction kits. A) Fragment of the *TPI* gene (60 bp) diluted to a concentration of 10 μg and run on agarose gel. B) Fragments purified and recovered from the agarose gel by the modified freeze-squeeze method and compared with those recovered by five commercial kits. C) Densitometry analysis performed for gel containing purified fragments. The error bars represent the standard deviation from three experimental replicates. M: Molecular weight marker (10 bp DNA Ladder, Thermo Scientific).

Furthermore, we compared the purification of PCR products obtained from the *TPI* gene using the freeze-squeeze modified method with five other commercial kits that indicated in their instruction manuals that fragments measuring 50 bp in length can be purified. However, the instructions also mentioned that for fragments measuring 50 bp in length, only 20% of the product is recovered. In order to test our modified freeze-squeeze procedure against these commercial kits, we performed a third experiment with the same 60-bp-long fragments (*TPI* gene) (Fig. 3A). DNA recovered from the bands extracted from the gel and purified with the modified freeze-squeeze method was compared to that recovered from the five commercial kits. In experiments using the kits, the bands were processed according to the instructions for each kit, and the elution volume of all samples, including the band processed by our modified freeze-squeeze method was 15 μL. The purified volume was then loaded and run on an agarose gel. Fig. 3B shows the products obtained with each of the commercial kits. We observed that commercial kit 1 did not recover the sample, while with commercial kits 2, 3 and 4, a faint and diffuse band on the new gel was obtained. Only the band treated with commercial kit 5 showed good purification, similar to that obtained with our modified freeze-squeeze method, where the recovered PCR product formed a well-defined and concentrated band. The densitometry analysis data obtained from this experiment confirmed that the modified freeze-squeeze method and commercial kit 5 were the best procedures to purify and concentrate the samples, compared to the other commercial kits that were used (Fig. 3C).

**Fig. 4:**
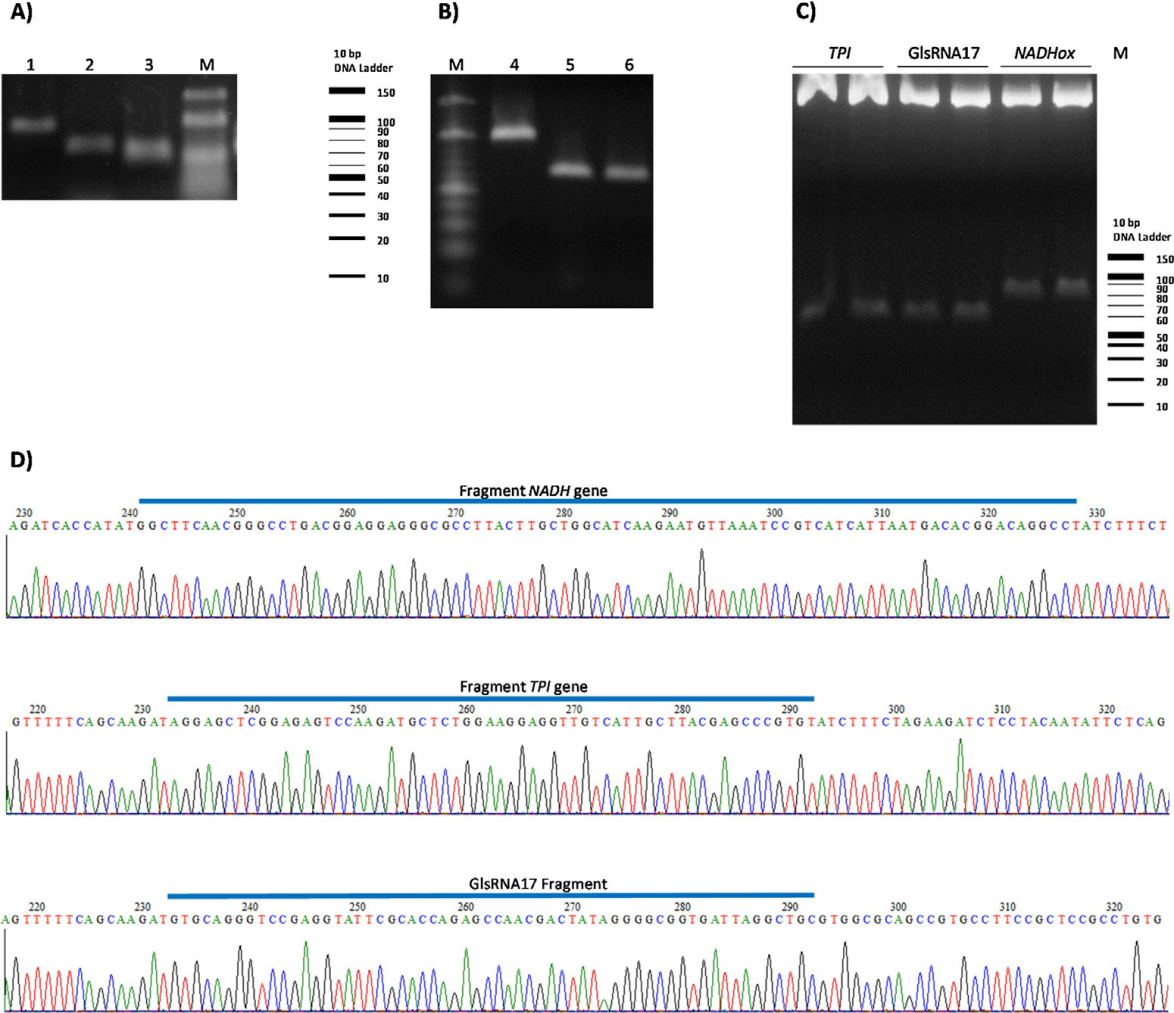
Applications of small DNA fragments purified from *G*. *lamblia*. A) Fragments corresponding to *NADHox* (98 bp), *TPI* (65 bp) genes and snoRNA GlsR17 (62 bp) (lines, 1, 2 and 3, respectively), were separated on 2.5% agarose gels. B) The three DNA fragments mentioned in (A) were recovered, diluted in 10 μL and then re-amplified again by PCR with a primer pair; for *NADHox* (Fw: GCACCATATGGCTTCAACGG and Rv: CAGGCCTGTCCGTGTCATTA); *TPI* (Fw: AGGAGCTCGGAGAGTCCAA and Rv: ACACGGGCTCGTAAGCAAT) and GlsRNA17 (Fw: TGCAGCCTAATCACCGC and GTGCAGGGTCCGAGGT). M: Molecular weight marker (10 bp DNA Ladder, Thermo Scientific). C) Blunt-end ligation of DNA fragments purified using the modified freeze-squeeze method and digestion of the fragments cloned into vector pJET1.2 with restriction enzymes with *Xho*I and *Nde*I D) Sequences obtained from the three fragments cloned into vector pJET1.2. Sequences analyses were carried out in the Unidad de síntesis y secuenciación del Instituto de Biotecnología, UNAM (Cuernavaca, México).

To demonstrate that the freeze-squeeze method with modifications is functional for fragments other than the *TPI* fragment, we performed experiments with other fragments of different sizes following the same purification protocol. We chose the 98 bp long gene encoding NADH oxidase (*NADHox*) and 62 bp of the small nucleolar RNA (snoRNA) GlsR17 (ID: AY820298.1), from which the mature microRNA miR2 of *G*. *lamblia* is derived [10], [11] (Fig. 4A). All DNA fragments analyzed were amplified by PCR using cDNA as the template, whereas the snoRNA fragments were obtained from samples of small RNAs extracted using the mirPremier^®^ microRNA Isolation Kit (Sigma-Aldrich). It is interesting to note that all samples obtained were used for other molecular applications as well. First, the purified samples were re-amplified by PCR; all extracted gel fragments were re-suspended in 10 μL and 1 μL was used as the DNA template to re-amplify the fragments and only 3 μL of the PCR product obtained was loaded, noting an increased concentration of each one of the bands (Fig. 4B). These results are important because the proposed method does not interfere with the re-amplification of PCR products and with this method, gene regions of low expression, such as miRNAs, can be easily recovered. Next, the remaining volume of the purified products was cloned into the pJET 1.2/blunt vector (CloneJET PCR Cloning Kit; Thermo Scientific), using T4 ligase (Thermo Scientific). Each construction was transformed into competent *Escherichia coli* Top 10F́ cells (Invitrogen™, USA), which were grown at 37 °C overnight on Luria Bertani (LB) agar plates supplemented with 100 μg/mL ampicillin. The plasmid DNA was extracted using the GeneJET Plasmid Miniprep Kit (Thermo Scientific) according to the manufacturer’s instructions. A total of 10, 19, and 36 colonies were obtained for *TPI*, *NADHox* and GlsRNA17, respectively. To confirm that the purified fragment had been inserted into the vector, two colonies from each construction were used to digest with restriction enzymes *Xho*I and *Nco*I (Thermo Scientific) (Fig. 4C). The purification method for small DNA fragments proposed in this work was also useful for cloning and sequencing of each of the sample fragments, which offers the advantage of identification and confirmation through nucleotide sequencing (Fig. 4C and 4D).

In summary, we proposed an alternative for the purification of small fragments of DNA by the freeze-squeeze method with some modifications, obtaining purified gene regions measuring 60–98 bp in length from standard agarose gels. These fragments were recovered without using agarose with a low melting point, membranes, resins, glycogen for precipitation and without commercial kits but by simple processes and basic solutions, which makes this technique an extremely practical and inexpensive methodology. With the obtained fragments, it was possible to have a higher DNA concentration and the products recovered were successfully used for other molecular applications, such as PCR or cloning. In addition, each purified fragment was cloned efficiently, making this technique beneficial for other molecular experiments such as sequencing, real-time PCR, isolation and identification of miRNAs, that are short-length fragments and difficult to manipulate but also of great importance in the gene regulation of *G*. *lamblia*, which is little studied.
